# Potential of infrared microscopy to differentiate between dementia with Lewy bodies and Alzheimer’s diseases using peripheral blood samples and machine learning algorithms

**DOI:** 10.1117/1.JBO.25.4.046501

**Published:** 2020-04-23

**Authors:** Ahmad Salman, Itshak Lapidot, Elad Shufan, Adam H. Agbaria, Bat-Sheva Porat Katz, Shaul Mordechai

**Affiliations:** a Shamoon College of Engineering, Department of Physics, Beer-Sheva, Israel; b Afeka Tel-Aviv Academic College of Engineering, Afeka Center for Language Processing, Department of Electrical and Electronics Engineering, Tel-Aviv, Israel; c Ben-Gurion University of the Negev, Department of Physics, Faculty of Natural Sciences, Beer-Sheva, Israel; d The Hebrew University of Jerusalem, School of Nutritional Sciences, The Robert H. Smith Faculty of Agriculture, Food, and Environment, Rehovot, Israel; e Kaplan Medical Center, Rehovot, Israel

**Keywords:** infrared spectroscopy, dementia with Lewy bodies, Alzheimer’s disease, WBC, plasma, machine learning

## Abstract

**Significance:** Accurate and objective identification of Alzheimer’s disease (AD) and dementia with Lewy bodies (DLB) is of major clinical importance due to the current lack of low-cost and noninvasive diagnostic tools to differentiate between the two. Developing an approach for such identification can have a great impact in the field of dementia diseases as it would offer physicians a routine objective test to support their diagnoses. The problem is especially acute because these two dementias have some common symptoms and characteristics, which can lead to misdiagnosis of DLB as AD and vice versa, mainly at their early stages.

**Aim:** The aim is to evaluate the potential of mid-infrared (IR) spectroscopy in tandem with machine learning algorithms as a sensitive method to detect minor changes in the biochemical structures that accompany the development of AD and DLB based on a simple peripheral blood test, thus improving the diagnostic accuracy of differentiation between DLB and AD.

**Approach:** IR microspectroscopy was used to examine white blood cells and plasma isolated from 56 individuals: 26 controls, 20 AD patients, and 10 DLB patients. The measured spectra were analyzed via machine learning.

**Results:** Our encouraging results show that it is possible to differentiate between dementia (AD and DLB) and controls with an ∼86% success rate and between DLB and AD patients with a success rate of better than 93%.

**Conclusions:** The success of this method makes it possible to suggest a new, simple, and powerful tool for the mental health professional, with the potential to improve the reliability and objectivity of diagnoses of both AD and DLB.

## 1 Introduction

Significant deterioration in the mental ability to deal with normal daily life is referred to as dementia. Alzheimer’s disease (AD) affects an estimated 60% to 80% of all people diagnosed with dementia and is considered to be the most common form of dementia among older people. Dementia with Lewy bodies (DLB) is thought to be the second most common form among the elderly,^1^^,^^2^ accounting for 20% of the cases at autopsy.^3^^,^^4^ It is estimated that, in the US alone, ∼10.5  million people have developed a type of dementia,^5^ with about 1.4 million of them suffering from DLB.^6^ Because AD and DLB are diseases related to aging, as life expectancy increases in the developed countries, these diseases become major ailments that lead to death and impose heavy financial burdens on both families and society.

It is relatively easy to identify a patient with dementia, but it is much more difficult to determine the type of dementia because its different forms have many overlapping symptoms mainly at the early stages.^7^ Early symptoms of both AD and DLB include difficulty remembering names and recent events, as well as depression. Later symptoms of AD include poor judgment, behavioral changes, and difficulty walking, speaking, and swallowing; these symptoms worsen with time.^3^ DLB patients, on the other hand, develop other symptoms, such as a blank expression, delusion, sleep disorders, decreasing alertness, recurrent visual hallucinations, fainting, fluctuations in autonomic processes, and repeated falls.^3^^,^^8^ A new study^9^ has reported that experts in the field of dementia diagnose AD clinically with a modest sensitivity of 71% to 87% and a specificity of 44% to 71%, when compared with the postmortem observations, which are considered the gold standard. The sensitivity of DLB’s consensus criteria^1^ is even lower than that of AD.^10^^,^^11^

The ability to clinically distinguish the different stages of AD and to track progression of the disease has been advanced via some recently developed biomarkers. These include neurofibrillary tangles,^12^ positron emission tomography (PET) ligands with high affinity for amyloid plaques,^13^ meta-analysis,^14^ and Amyloid Imaging Taskforce.^15^^,^^16^ The most accepted imaging methods commonly used for diagnosing both AD and DLB are magnetic resonance imaging (MRI), single photon emission computed tomography (CT), CT scanning, and PET.^17^ The use of cerebrospinal fluid (CSF) and neuroimaging to obtain specific biomarkers of the disease has accelerated in recent years, despite their high cost and their frequent unavailability.^11^^,^^18^ Many patients have both diseases; as a result, the MRI and amyloid markers become less discriminative.^11^^,^^19^

It is highly important to accurately diagnose the type of dementia at the early stages of AD and DLB,^17^ even though there are no specific medications to treat these neurological diseases. For example, early diagnosis of DLB can prevent the side effects of the neuroleptic drugs^20^ often given to AD patients and improve the response to cholinesterase inhibitors.^21^ Moreover, early and objective diagnosis enables targeted treatments that lead to deceleration of the rate of increase of symptoms of these dementias, which can lead to longer-term improvement of the patient’s quality of life.^11^^,^^22^

Currently, the diagnosis of AD and DLB relies on an evaluation of the medical history of the patient in addition to the physical and laboratory examinations, which include tests of blood components^16^ and the methods mentioned above.^23^

The use of IR spectroscopy for medical diagnostics is in a period of significant acceleration, as it offers an accurate, inexpensive, and rapid method of analysis that has been widely used for various medical purposes for about 30 years. For example, IR has been used for classification of different kinds of cancers and infectious diseases^24^^–^^35^ by analyzing tissue samples and biofluids.^24^^,^^25^^,^^28^^,^^31^^,^^34^^–^^39^

An interesting recent study has demonstrated the ability to detect AD based on changes in the amide I protein’s secondary structure due to amyloid beta conformation,^37^ which can be monitored by IR. Recent studies have demonstrated the potential of FTIR microscopy and Raman spectroscopy accompanied with multivariate analysis for the detection of AD through the analysis of plasma^40^^–^^42^ and white blood cells (WBC).^43^^,^^44^ In the present study, we take this further, showing for the first time the potential of IR spectroscopy of WBC and plasma in tandem with machine learning classifiers to enable a differential diagnosis of DLB and AD patients.

## 2 Materials and methods

### 2.1 Preparation of Samples

This study was carried out with the approval of the Institutional Review Board (Helsinki Committee) and with the consent of the dementia patients or their guardians. The physicians diagnosed the cohort as controls, AD patients, and DLB patients using the classical methods, which are based on the evaluation of the patients’ medical histories; physical and laboratory imaging examinations such as MRI, CT, and PET; and the personal experience of the physicians who treated these patients for a long period of time. Blood samples were collected and analyzed to fulfill the objectives of the current study. WBC and plasma components were separated from whole blood samples (2 to 3 mL) within <3  h after collection. The Hudson and Poplack method was followed to accomplish the separation process.^45^ Briefly, 3 mL of Histopaque solution (Sigma Chemical Co., St. Louis, Missouri) was added to the blood tube before being centrifuged at 300 g for 30 min at 23°C. After centrifuging, the WBC, which appears as a layer located at the middle of the tube, was isolated and washed with phosphate-buffered saline by centrifugation at 300g for 10 min at 23°C. One microliter of WBC samples and the same amounts of plasma samples were mounted as separated drops on zinc selenide crystal, which is transparent to IR radiation. The samples were dried under laminar flow for about 15 min before measuring.

### 2.2 FTIR Measurements and Spectral Processing

All measurements were obtained using an Equinox 55 spectrometer made by BRUKER, Germany, that was coupled to an IR microscope with an MCT detector. All measurements were performed in the 600- to $4000\text{-}{\mathrm{cm}}^{-1}$ spectral region with the following specifications: spectral resolution $4\;{\mathrm{cm}}^{-1}$, transmission mode, and 128 co-added scans. The measured spot was determined to be a circle 100-μm in diameter. The time needed to perform the 128 scans is about 80 s. At least five different spots were measured from each sample.

The processing of the spectra was done using OPUS 7 software of BRUKER, Germany. The processing procedure included smoothing, bisecting the measured region to 950 to $1760\;{\mathrm{cm}}^{-1}$, baseline correction, and normalization, as reported in our previous studies.^43^^,^^46^

The available details, age, gender, and dementia type of the patients included in this study are listed in Table S1 in the Supplementary Material. Two blood components, plasma and WBC, were separately chosen to characterize each patient. The AD category was further defined to include the three stages of the disease, namely mild, moderate, and severe AD cases.

Table 1 summarizes the data from Table S1 in the Supplementary Material, listing the number of patients and the number of spectra acquired for each investigated category.

**Table 1:** The number of patients and number of spectra for each investigated category.

Sample type	Controls		AD		DLB		Dementia (AD and DLB)	
	No. of patients	No. of spectra	No. of patients	No. of spectra	No. of patients	No. of spectra	No. of patients	No. of spectra
WBC	26	145	20	121	10	59	30	180
Plasma	23	111	18	87	8	39	26	126

Infrared (IR) microscopy spectroscopy was used to examine WBC and plasma isolated from 56 individuals: 26 controls (14 females and 10 males), 10 DLB (8 females and 2 males), and 20 AD patients (14 females and 10 males). The measured spectra were analyzed via machine learning.

### 2.3 Machine Learning Analysis

The preprocessed raw data are the IR absorption spectra of the WBC or plasma. This data can be used as high-dimensional feature vectors. Each of the feature vectors consists of 451 dimensions of different wavenumbers (wavelengths). Some of these wavenumbers do not carry any additional specific information to other wavenumbers for the classification task; thus different methods are used for efficient feature selection to exclude these wavenumbers from the original features vectors before training the classifiers.^47^^–^^51^ The aim is to find a low-dimensional representation of the data to increase classifier performance.

As has been found in previous works,^51^^,^^52^ the second derivative of the raw feature vector is much more informative and leads to a much better classification, so the feature selection was performed on those vectors.

**Fig. 1:**
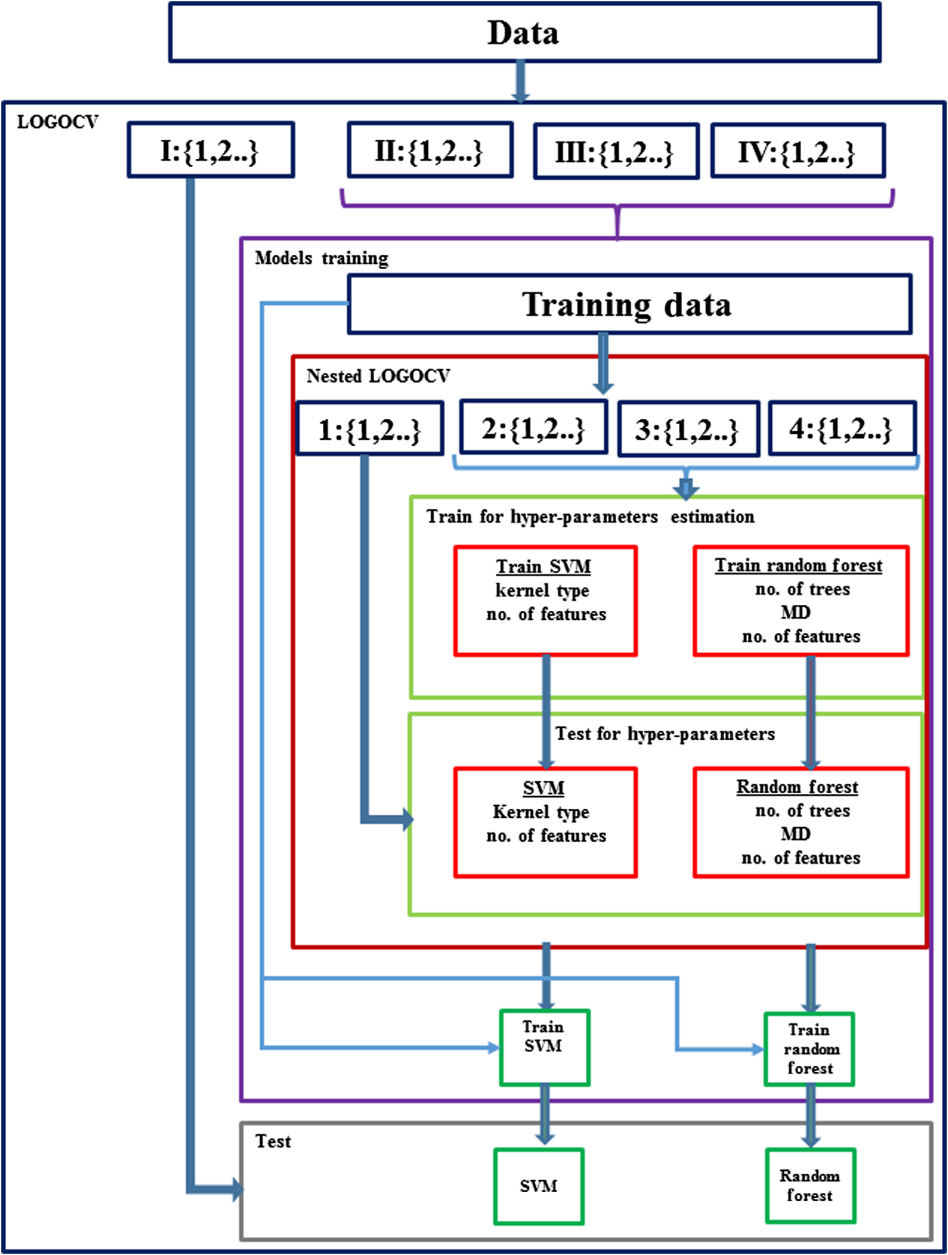
Schematic description of the training and testing process, including the different classifiers, feature selection, and hyper-parameters tuning.

To obtain the optimal hyper parameters of each of the tested classifiers, a nested cross validation was applied.^53^ Figure 1 summarizes schematically all of the major processes used for developing the classifiers used.

### 2.4 System Description

The overall system is shown in Fig. 1. As the number of patients is small, we validate the system using leave-one-out on a patient’s level. All of the feature vectors of one patient are taken out for testing, and all other data (individual spectra of the other patients) is used for training. We name this procedure as leave one group out cross validation (LOGOCV). In this analysis, each patient was considered a group for WBC and plasma samples. Therefore, all of the spectra except for one group (spectra belonging to the same patient) were used for training the classifier, and then the excluded group was used for validation, spectrum by spectrum. The diagnosed category of the specific patient (group) was determined by voting on the diagnosed categories for each of the spectra belonging to this patient. This procedure was repeated as many times as the number of patients included in each experiment. To define the hyper-parameters of the system, due to the lack of data we apply a nested cross validation, again using LOGOCV. From the training dataset, one group (patient) is taken out and the system is trained using different numbers of selected features and different classifiers’ parameters [kernel type for support vector machine (SVM); number of trees and the depth of the trees for random forest (RF)]. After the optimal hyper parameters are set, that chosen classifier is trained and tested on the one group that was out. The process is repeated for every patient in the database.

Tuning of the hyper parameters, number of trees and maximum depth (MD) for the RF and kernel type of the SVM, was applied before training. For both stages of classification, the number of trees for the WBC was 10 to 20 while for plasma it was 10 to 15. The MD for both the WBC and plasma was 2 to 5. For all classifications using SVM, the third-order polynomial kernel gave the best results, except for the classification between AD-controls using WBC in which the radial basis function (RBF) kernel gave the best classification results.

### 2.5 Features Selection

**Fig. 2:**
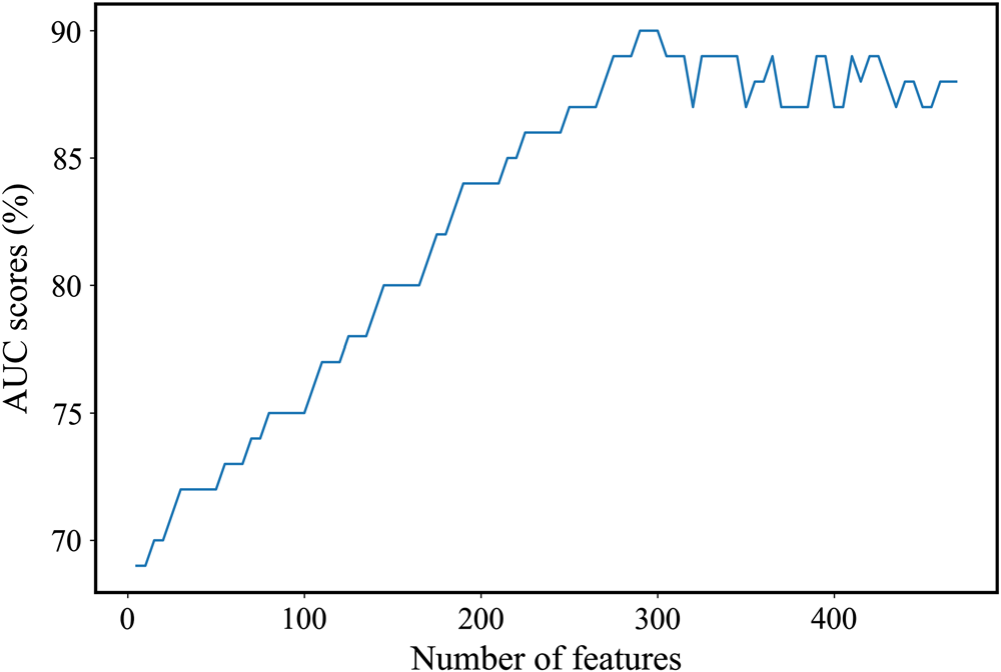
AUC scores in percentage versus the number of selected features of the second derivative spectra of WBC in the 900- to $1800\text{-}{\mathrm{cm}}^{-1}$ region for the SVM classifier for the classification between dementia and controls.

The chi-square method was used in this study for feature selection. This method calculates the independence of two categories^54^ based on the same feature (wavenumber) of all of the features that appear in the original feature vector. Then these features are arranged in descending order based on the scores calculated by the chi-square; the first feature thus has the highest score and hence is the most discriminative feature. The performance of the desired classifier is evaluated using nested cross validation based on different new feature vectors, which contains selected features (wavenumbers) of the arranged features. The first new feature vector contains the first five most discriminative wavenumbers; the second contains the first ten most discriminative wavenumbers and so on. This procedure is repeated about 90 times; each time the dimension of the new feature vector is increased by five.^54^ The area under curves (AUCs) of the SVM classifier versus the number of selected features is plotted in Fig. 2. The selected features are those included in the new feature vector that enables the classifier to achieve the highest AUC score. As can be seen from Fig. 2, when 300 features are used, the classifier achieves the highest accuracy. Similar figures were generated to select the optimal number of features for the different classifiers and different experiments.

For example, in Table S2 in the Supplementary Material, we list the top 50 selected features (wavenumbers) of the second derivative spectra of WBC in the 900- to $1800\text{-}{\mathrm{cm}}^{-1}$ region adapted in our analysis for the SVM classifier for the classification between dementia and controls.

### 2.6 Classifiers

We built a different RF^47^ and SVM^55^^,^^56^ for the classifications among the different categories, dementia (combined AD and DLB) and controls, AD and controls, DLB and controls in the first stage, and AD and DLB in the second stage. The measured spectra of WBC and plasma were used to discriminate between AD, DLB, and controls.

#### 2.6.1 SVM

The SVM classifier is a discriminative algorithm. It does not build a model for each class, but only finds the discriminative hyperplane with the largest margin determined by the support vectors from the training data.^57^ SVM is a linear classifier, usually used in the high-dimensional space (possibly infinite dimension) defined by the used kernel. Several kernels, polynomials of the first, second, and third order, and RBF, were under examination via nested cross validation.

#### 2.6.2 Random forest

The RF’s decision trees were constructed based on different training subsets chosen randomly from the original data (training data), with replacement, using a bootstrap sample.^58^^,^^59^ The reduced dimension trees (classifiers) were used to determine the category of the validation spectra; thus the prediction is more accurate.

### 2.7 Statistical Parameters

In this work, a two-stage strategy was used for the classification. In the first stage, the classification was performed between controls versus dementia (combined AD and DLB), controls versus DLB, and controls versus AD. In the second stage, we classify AD versus DLB. In all of the experiments, we used the WBC and plasma data in the 950- to $1760\text{-}{\mathrm{cm}}^{-1}$ low-wavenumber region separately.

Our problem was one of the binary classifications. In our analysis in the first stage, we defined AD, DLB, or dementia as a “positive” state and the controls as a “negative” state, whereas in the second stage, the AD was defined arbitrarily as the “positive” state and the DLB as the “negative” state. Different statistical measures were used to estimate the performances of the classifiers: true positive (TP) is the number of true predicted positive state samples; true negative (TN) is the number of true predicted negative state samples; false positive (FP) is the number of false predicted positive state samples; and false negative (FN) is the number of false predicted negative state samples. We calculated the performances in terms of accuracy, sensitivity, specificity, positive predicted value (PPV), and negative predicted value (NPV) as follows:

$$\text{accuracy}=\frac{\mathrm{TP}+\mathrm{TN}}{\mathrm{TP}+\mathrm{TN}+\mathrm{FP}+\mathrm{PN}};\;\text{sensitivity}=\frac{\mathrm{TP}}{\mathrm{TP}+\mathrm{FN}};\;\text{specificity}=\frac{\mathrm{TN}}{\mathrm{TN}+\mathrm{FP}};\;\mathrm{PPV}=\frac{\mathrm{TP}}{\mathrm{TP}+\mathrm{FP}};\;\mathrm{NPV}=\frac{\mathrm{TN}}{\mathrm{TN}+\mathrm{FN}}.$$

## 3 Results

**Fig. 3:**
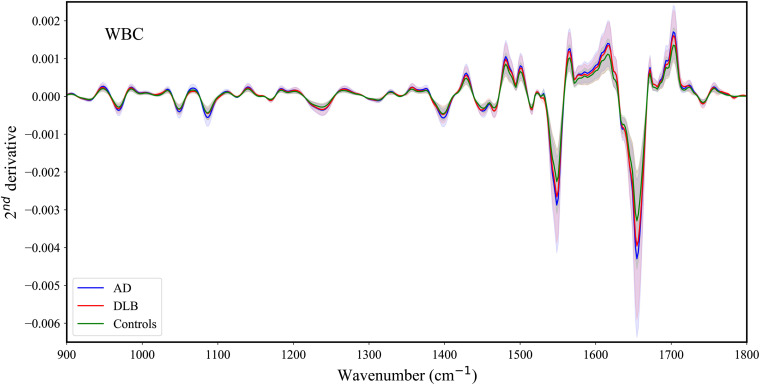
WBC IR second derivative average spectra of DLB, AD, and controls in the 900-$1800\;{\mathrm{cm}}^{-1}$ region. The highlighted areas represent the standard deviation of the spectra within each category.

The mid-IR spectra are considered fingerprints of the samples. These spectra are related to the functional groups of the biomolecules that compose the measured samples, proteins, lipids, nucleic acids, and carbohydrates. The spectra were used quantitatively by applying supervised multivariate analysis for the purpose of differentiation. Figure 3 displays the average second derivative spectra of the WBC blood component for AD and DLB, in the 900- to $1800\text{-}{\mathrm{cm}}^{-1}$ range (the IR average spectra are plotted in Fig. S1 in the Supplementary Material). The AD category includes all three subgroups (mild, moderate, and severe) of the stages of the disease. The signatures of all of the biomolecules appear in the spectra.

Table 2 summarizes the functional groups associated with major vibrational bands in the second derivative spectra of WBC shown in Fig. 3.^60^^–^^73^

**Table 2:** Assignments of the functional groups in the IR spectra.

Wavenumber (${\mathrm{cm}}^{-1}$)	Molecular vibrations of the functional groups and biomolecule contributor
1741	Phospholipids are the main contributors
1590 to 1727	Amide I absorption bands (mainly proteins)
1480 to 1590	Amide II absorption bands (mainly proteins)
1395	Proteins, lipids, and amino acids are the main contributors
1200 to 1340	Amide III (mainly proteins)
1185 to 1485	Contributed mainly by phosphate, proteins, nucleic acids, and lipids
950 to 1185	Carbohydrates are the main contributors

**Fig. 4:**
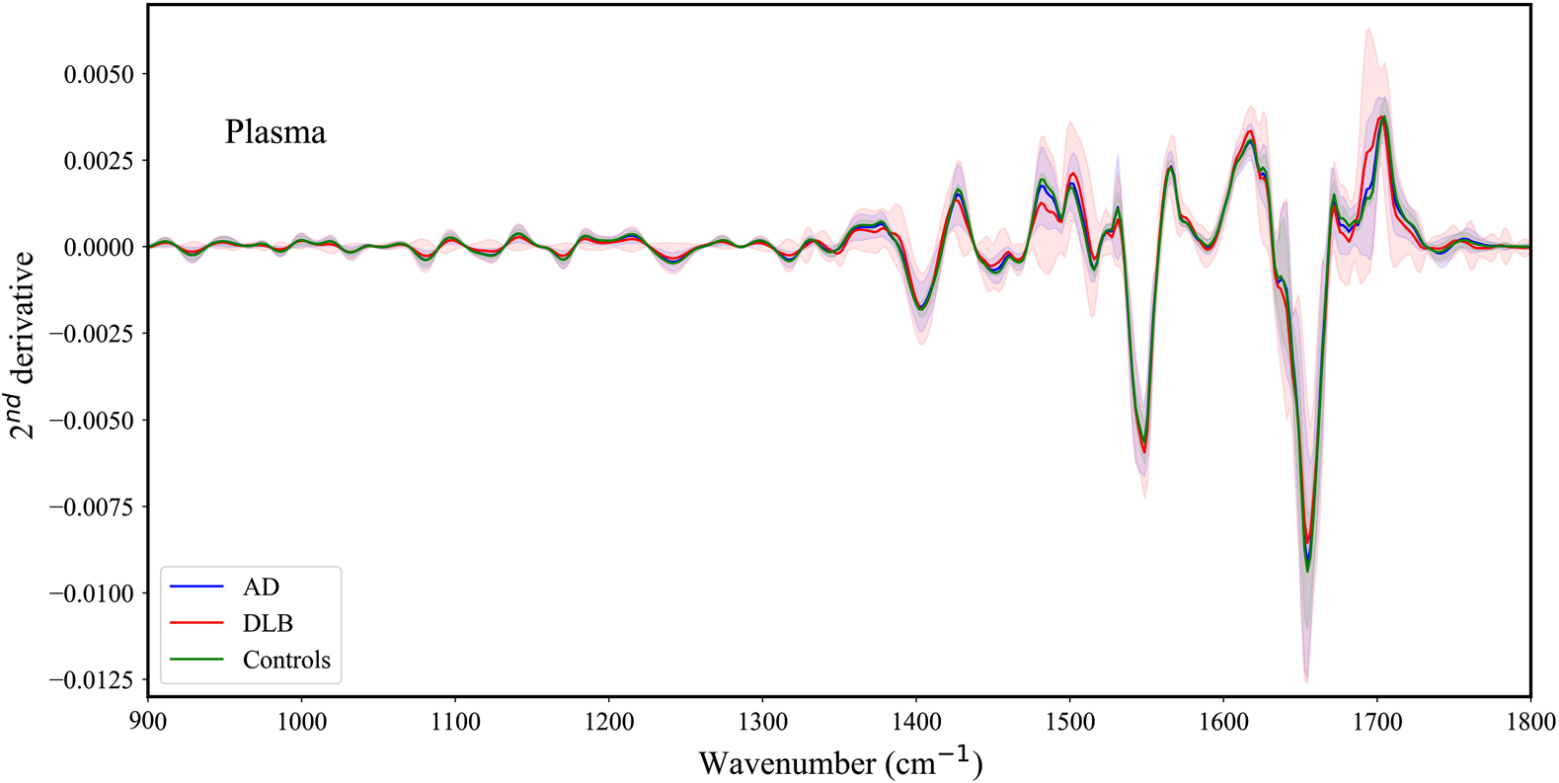
Plasma IR second derivative average spectra of DLB, AD, and controls in the 900-$1800\;{\mathrm{cm}}^{-1}$ region. The highlighted areas represent the standard deviations of the spectra within each category.

As can be seen from Fig. 3, the spectral differences among the three groups are minute. Similarly, the average second derivative IR absorption spectra of plasma obtained from AD and DLB patients are presented in Fig. 4 (the IR average spectra are plotted in Fig. S2 in the Supplementary Material).

The spectral differences among the three groups are minute (Figs. 3 and 4), so we applied multidimensional machine learning algorithms to differentiate between the various categories.

The potential of the SVM and RF algorithms for classification purposes in similar cases has been previously well established.^51^^,^^74^^,^^75^ Here although the spectral differences were subtle, they were still repeatable enough to achieve a good classification, as is shown below.

We considered a binary classification problem with spectra from blood components being grouped based on their categories as dementias or controls using SVM and RF.^51^^,^^55^^,^^56^^,^^58^^,^^59^ For this analysis, we focused on the low-wavenumber spectral region (950 to $1760\;{\mathrm{cm}}^{-1}$); LOGOCV was used for the optimization of the parameters of the classifiers and estimation of their performances.

Many experiments were run to differentiate among the different categories included in each experiment to estimate the performance of the system. The performances of the SVM and RF developed classifiers for their optimal configurations (achieved using nested cross validation) were evaluated using the receiver operating characteristic (ROC) curves. The AUC represents the quality of the classifier. For each experiment, we determined the performance of the best classifier, the best system that enables the best classification results, using the following statistical terms: SP, SE, Acc, PPV, NPV, and AUC.

**Fig. 5:**
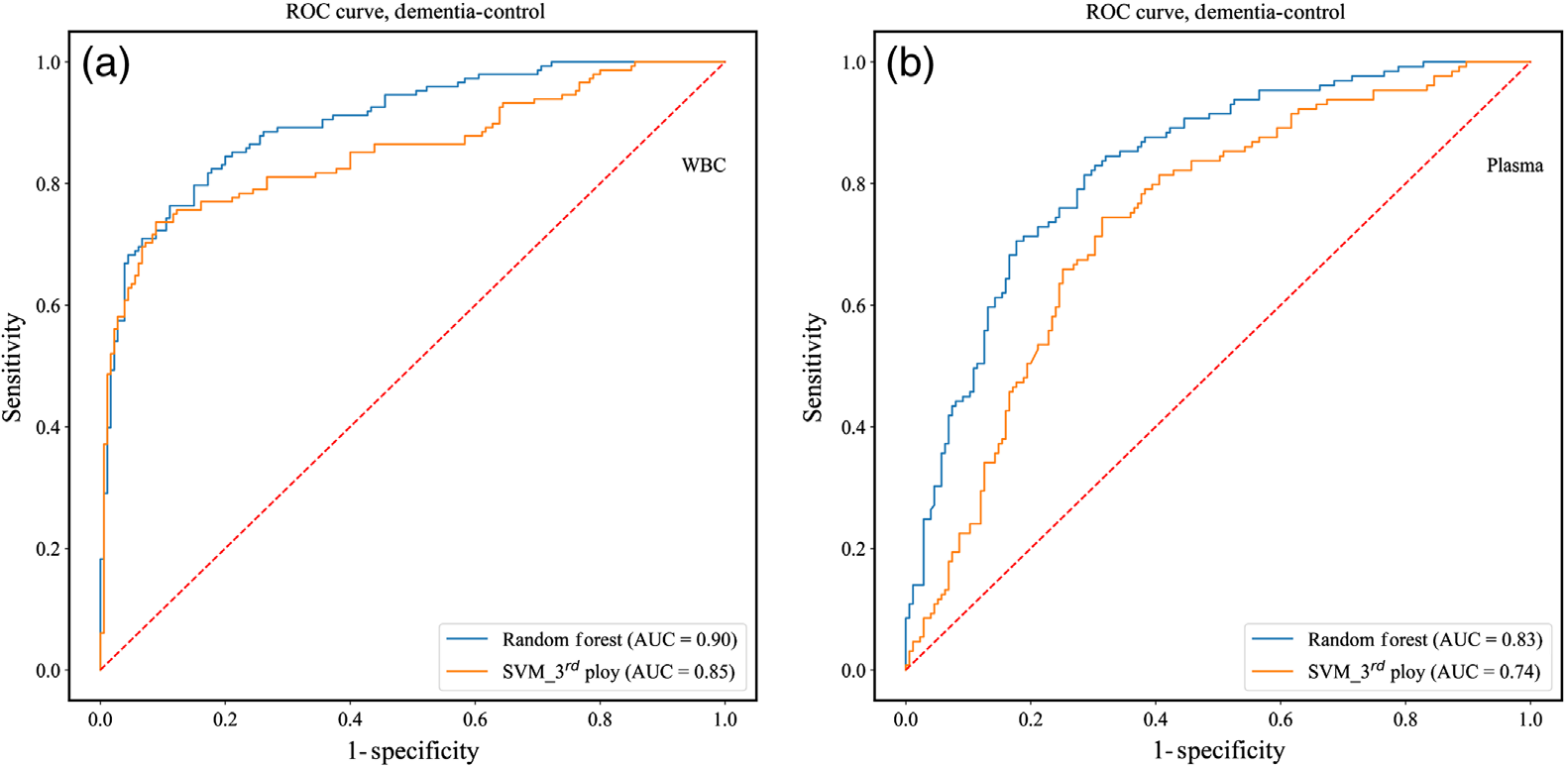
Resulting ROC curves of the different classifiers for the classification between dementia and controls categories using selected features from the FTIR second derivative spectra, in the 900-$1800\;{\mathrm{cm}}^{-1}$ region, for the two blood components: (a) WBC and (b) plasma. The curves scores were derived at the spectrum level using the LOGOCV approach for both classifiers.

Figure 5 shows the ROC curves for the differentiation between dementia (combined AD and DLB) and controls using selected features of the second derivative spectra of the two blood components, WBC and plasma, separately. The curves scores were derived at the spectrum level using the LOGOCV approach. The classification results, derived at the patient level by voting on the results of the second derivative spectra belonging to the specific patient, are summarized in Table 3.

**Table 3:** Performances of the best-used-classifier for the classification between dementia and controls categories. The classification results were computed at the patient level by voting the results of the classifier at the spectrum level, derived using the LOGOCV approach, for all of the feature vectors that belong to the specific patient.

	Best classifier	No. of features	SE	SP	Acc	PPV	NPV	AUC
WBC	RF	300	0.90	0.81	0.86	0.84	0.88	0.90
Plasma	RF	300	0.81	0.80	0.81	0.82	0.79	0.83

Similar analyses were done for the AD-controls and DLB-controls (Figs. S3, S4 and Tables S3, S4 in the Supplementary Material).

As can be seen from Table 3, each of the blood components gives good results. However, the WBC samples gave superior classification results when compared with the plasma samples.

The same behavior can be seen in Tables S3 and S4 in the Supplementary Material. Each of the blood components gives reasonable results. However, the WBC samples classification results are again better when compared with the plasma samples.

In the second stage, two experiments were performed to differentiate between AD and DLB categories using selected features of the second derivative spectra of the two blood components, WBC and plasma, separately.

**Fig. 6:**
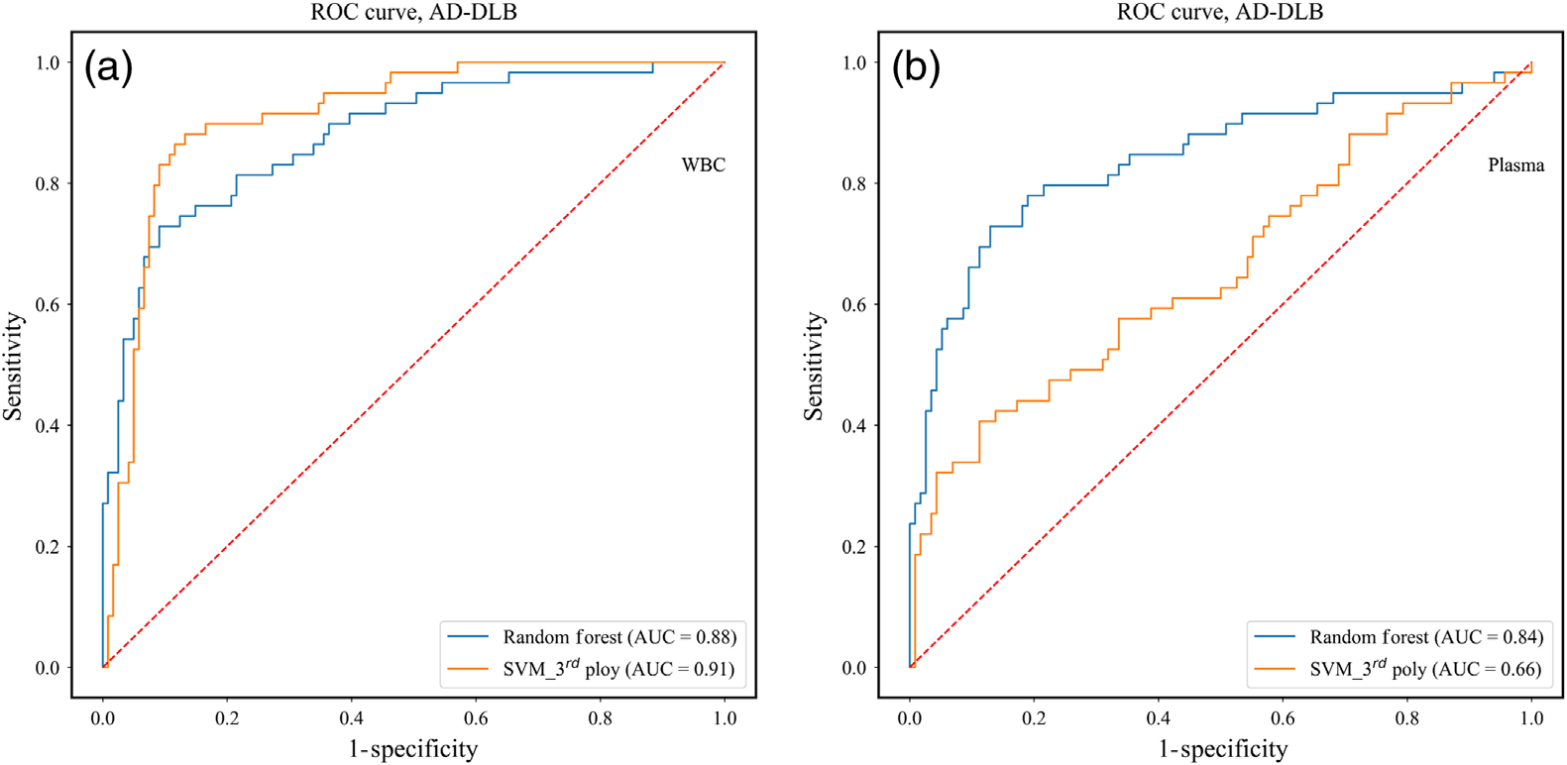
Resulting ROC curves for the different classifiers for the classification between AD and DLB categories using selected features from FTIR second derivative spectra, in the 900-$1800\;{\mathrm{cm}}^{-1}$ region, for the two blood components: (a) WBC and (b) plasma. The curves scores were derived at the spectrum level using the LOGOCV approach for both classifiers.

Figure 6 shows the ROC curve for the two experiments of the above second stage: (a) WBC AD-DLB and (b) plasma AD-DLB. The curves scores were derived at the spectrum level using the LOGOCV approach. The results of the classifications for the two experiments, derived at the patient level by voting the results of the second derivative spectra belonging to the specific patient, are summarized in Table 4.

**Table 4:** Performances of the best used classifier for the classification between AD and DLB categories. The classification results were computed at the patient level by voting the results of the classifier at the spectrum level, derived using the LOGOCV approach, for all of the feature vectors that belong to the specific patient.

	Best classifier	No. of features	SE	SP	Acc	PPV	NPV	AUC
WBC	SVM third poly	310	0.95	0.90	0.93	0.95	0.80	0.91
Plasma	RF	300	0.83	0.75	0.81	0.91	0.60	0.84

A success rate higher than 90% was achieved based on the WBC data. The WBC classification results are again superior relative to plasma.

To evaluate the potential of our system for the classification between early stages of AD patients and controls or DLB, additional experiments were performed. In these additional experiments, the AD patients were subdivided into the mild, moderate, and severe stages using the SVM classifier. We tried to differentiate between the different pairings: DLB-AD moderate, DLB-AD mild, DLB-AD severe, DLB-combined mild and moderate, controls-AD moderate, controls-AD mild, and controls-AD as described in Table 5. The performances of the SVM classifiers were derived based on WBC data using selected features of the second derivative spectra.

**Table 5:** Performances of the SVM classifier in percentage for the classification between the different couples of categories, controls, DLB, and the three stages of AD, mild moderate, and severe. The classification results were computed based on the WBC data at the patient level by voting the results of the classifier at the spectrum level, derived using the LOGOCV approach, for all of the feature vectors that belong to the specific patient.

Category pairing	AUC	ACC	SE	SP	PPV	NPV
DLB-AD moderate	0.81	0.87	0.75	0.9	0.5	0.96
DLB-AD mild	0.91	0.97	0.75	1	1	0.96
DLB-AD severe	0.92	0.91	1	0.8	0.86	1
DLB-AD combined mild and moderate	0.94	0.83	0.88	0.8	0.78	0.89
Controls-AD mild	0.75	0.86	0.75	0.81	0.38	0.95
Controls-AD moderate	0.9	0.87	0.75	0.88	0.5	0.96
Controls-AD severe	0.95	0.96	0.92	1	1.0	0.96
DLB-AD combined mild and moderate	0.88	0.85	0.87	0.8	0.67	0.92

The number of patients included in the analysis is as follows: 26 controls, 10 DLB patients, 4 AD moderate, 4 AD mild, and 12 AD severe.

## 4 Discussion

The analysis of biofluids such as serum and urine, which is considered minimally invasive, low risk^76^ and inexpensive,^36^ is promising as a future healthcare tool for the diagnosis of different diseases.^76^ There is a clear need for a new method that should be sensitive, objective, reliable, and effective for screening very large numbers of people, one that can be used after full development as a simple routine test^77^ to help the mental health professional improve the reliability and objectivity of the diagnoses of both AD and DLB. Our study shows promise of a method that can reach this goal by combining IR spectroscopy of WBC and plasma—which can be isolated from peripheral blood with relative ease^78^—with advanced machine learning methods.

A recent study^79^ reported the use of a machine learning classifier, RF, to improve the accuracy of the diagnosis between AD and DLB patients based on electroencephalography.

In previous studies, we used the IR spectra of WBC and plasma to differentiate directly between DLB and controls and between AD and controls.^43^ In this study, our goal was to investigate the potential of IR spectroscopy for differentiating between AD and DLB. Our results, based on a limited number of assessments, show that it is possible to differentiate between dementia (AD and DLB) and controls with a success rate that exceeds 86% (Fig. 5 and Table 3). In the second stage in which the aim was to differentiate between AD and DLB, a success rate that exceeds 90% was achieved (Fig. 6 and Table 4).

The biochemical changes in the blood components associated with developing AD and DLB are minute, and this is reflected in the minor spectral changes among the WBC and plasma of the different categories. To monitor the spectral changes among the different categories, it is very important to measure high signal-to-noise ratio and reproducible spectra. Thus we used the transmission sampling technique and measured at least five spectra from different sites of the same sample. To examine the reproducibility of the spectra, an overlay of five spectra that were acquired from different sites of the same WBC sample were plotted in Fig. S5 in the Supplementary Material. The spectra almost completely overlay each other, indicating an excellent reproducibility.

SVM and RF are supervised methods, so they should be trained before validation. The training spectra were determined using the physicians’ “gold standard prognoses” of these neurological diseases after an extended follow-up of the patients included in this study. Yet the physicians’ prognoses also have a limited confidence level. These uncertainties in the physicians’ diagnoses contribute to classification errors. As the accumulated incidence of both AD and DLB neurological diseases is about 85%^80^^,^^81^ of all dementia patients, the ability to differentiate between these two major forms of the disease is highly important.

The success of the development of this method will have a great impact in the field of dementia diseases as it could offer physicians a routine objective test to support their diagnosis.

Many methods have been studied for the diagnosis of AD, but each has major drawbacks. For example, the CSF biomarkers are not standardized.^82^ The diagnostic value of neuroimaging, genetic, and biochemical biomarkers developed for AD has as yet not been established by vigorous testing. Moreover, many of the developed techniques are difficult to perform due to expensive instrumentation and/or reagents or due to potential hazards, as is the case with CSF extraction.^77^

The use of pattern recognition methodologies and sophisticated multivariate statistical tools in the field of medicine is achievable now due to the development of modern IR spectrometers, advanced computers, and powerful new algorithms.^73^^,^^79^

As can be seen from Figs. 5–6 and Tables 3–4, analyzing the IR absorption spectra acquired from WBC and plasma blood components shows great promise as a method for differentiating among the three categories of controls, AD, and DLB.

The machine learning classifiers were used for the prediction of AD (combined mild, moderate, and severe) and DLB or controls. Combining the three stages of AD in one category increases the heterogeneity in the AD category, resulting in a much more difficult situation for the classifier. Even so, the system has achieved good classification rates as can be seen from Tables 3 and 4, as well as Tables S3 and S4 in the Supplementary Material.

Although based on a limited number of patients, our results, summarized in Table 5, show that the system has a good potential for reliably diagnosing the AD type of dementia at its early stages. Enlarging the database will make the conclusions more reliable. As the number of cases of the mild and moderate AD stages is low (eight cases altogether), our analysis focused on the SVM classifier, which is more suitable for handling small databases.

The spectral changes that are spread over the entire spectral region cannot be related with high specificity to the exact biochemical changes that accompany the initiation of the neurological disease. Nonetheless, the differentiation between DLB and AD, which is the main goal of this study, is still a very important achievement in neurology.

## 5 Conclusions

Our technique, a combination of IR spectroscopy and machine learning, allows differentiation between DLB and AD with a high rate of success, with results obtained within ∼30 min of collection of a blood sample from a patient. This method may provide a new and significant tool by which health professionals can improve their diagnostic accuracy in distinguishing between AD and DLB patients beyond the level available from currently used methods. Our technique has a clear advantage for such diagnoses because it is simple, minimally invasive (compared with CSF and other imaging methods), and suitable for screening of samples on a large scale.

The classification results based on WBC samples were found to be superior to those obtained from the plasma samples.
