# Supplementary material for: Potential of infrared microscopy to differentiate between dementia with Lewy bodies and Alzheimer’s diseases using peripheral blood samples and machine learning algorithms
Source: J Biomed Opt. 2020 Apr 23;25(4):046501. doi: 10.1117/1.JBO.25.4.046501 (PMC7177186; doi:10.1117/1.JBO.25.4.046501)

**Table 1S:** Patients and measurement details of each of the investigated categories included in this study.

| **Serial No.** | **Type of dementia** | **Sex** | **Age** |
| --- | --- | --- | --- |
| 1 | AD-Mod | F | 82 |
| 2 | AD-Mild | F | 71 |
| 3 | DLB | F | 89 |
| 4 | DLB | M | - |
| 5 | Control | F | 77 |
| 6 | Control | F | 75 |
| 7 | AD-Severe | M | 78 |
| 8 | AD-Severe | F | 81 |
| 9 | DLB | F | 80 |
| 10 | DLB | F | 88 |
| 11 | Control | F | 78 |
| 12 | Control | F | - |
| 13 | DLB | F | 52 |
| 14 | AD-Mild | F | - |
| 15 | Control | M | 60 |
| 16 | Control | F | 91 |
| 17 | AD-Severe | F | 76 |
| 18 | AD-Severe | M | 80 |
| 19 | DLB | M | 82 |
| 20 | DLB | f | 82 |
| 21 | Control | f | 56 |
| 22 | Control | M | 51 |
| 23 | Control | F | 60 |
| 24 | AD-Mild | M | 75 |
| 25 | AD-Mod | M | 72 |
| 26 | AD-Mod | F | 60 |
| 27 | Control | F | 73 |
| 28 | Control | F | 75 |
| 29 | Control | M | 94 |
| 30 | Control | M | 81 |
| 31 | Control | M | 81 |
| 32 | AD-Severe | M | 90 |
| 33 | AD-Severe | M | 79 |
| 34 | Control | M | 81 |
| 35 | Control | F | 75 |
| 36 | Control | F | 77 |
| 37 | AD-Severe | F | 72 |
| 38 | DLB | F | 77 |
| 39 | Control | F | 75 |
| 40 | Control | M | 81 |
| 41 | AD-Severe | M | 60 |
| 42 | AD-Mod | F | 60 |
| 43 | DLB | F | 88 |
| 44 | Control | M | - |
| 45 | AD-Severe | F | 71 |
| 46 | DLB | F | - |
| 47 | Control | M | - |
| 48 | AD-Severe | M | 83 |
| 49 | Control | F | 77 |
| 50 | Control | F | - |
| 51 | AD-Mild | M | 72 |
| 52 | AD-Mod | F | 79 |
| 53 | Control | M | 82 |
| 54 | AD-Mod | F | 73 |
| 55 | AD-Severe | M | 83 |
| 56 | Control | F | 92 |

**Table 2S:** The top 50 selected features of the second derivative spectra of WBC in 900-1800 cm^-1^ region sorted in descending order based on their Chi-square scores, used for the classification between dementia and controls.

| **Serial No.** | **Selected features (wavenumbers cm^-1^)** | **Chi-square scores** | **Serial No.** | **Selected features (wavenumbers cm^-1^)** | **Chi-square scores** |
| --- | --- | --- | --- | --- | --- |
| 1 | 1001 | 0.2559 | 26 | 1766 | 0.2325 |
| 2 | 999 | 0.2550 | 27 | 984 | 0.2325 |
| 3 | 1003 | 0.2536 | 28 | 985 | 0.2323 |
| 4 | 997 | 0.2516 | 29 | 962 | 0.2321 |
| 5 | 993 | 0.2493 | 30 | 1778 | 0.2318 |
| 6 | 991 | 0.2487 | 31 | 978 | 0.2305 |
| 7 | 995 | 0.2486 | 32 | 974 | 0.2298 |
| 8 | 1005 | 0.2481 | 33 | 1011 | 0.2281 |
| 9 | 1786 | 0.2455 | 34 | 960 | 0.2280 |
| 10 | 1007 | 0.2428 | 35 | 976 | 0.2278 |
| 11 | 989 | 0.2413 | 36 | 1776 | 0.2241 |
| 12 | 1784 | 0.2409 | 37 | 958 | 0.2239 |
| 13 | 1788 | 0.2391 | 38 | 1763 | 0.2222 |
| 14 | 966 | 0.2364 | 39 | 1768 | 0.2202 |
| 15 | 1009 | 0.2363 | 40 | 957 | 0.2199 |
| 16 | 968 | 0.2360 | 41 | 1012 | 0.2196 |
| 17 | 970 | 0.2348 | 42 | 1790 | 0.2178 |
| 18 | 1782 | 0.2345 | 43 | 955 | 0.2165 |
| 19 | 987 | 0.2343 | 44 | 953 | 0.2132 |
| 20 | 964 | 0.2342 | 45 | 1014 | 0.2089 |
| 21 | 982 | 0.2329 | 46 | 951 | 0.2083 |
| 22 | 972 | 0.2329 | 47 | 1761 | 0.2075 |
| 23 | 1765 | 0.2326 | 48 | 1774 | 0.2066 |
| 24 | 980 | 0.2326 | 49 | 1770 | 0.2016 |
| 25 | 1780 | 0.2326 | 50 | 949 | 0.1994 |

**Fig. 1S:** WBC IR average spectra of DLB, AD patients, and controls in the 900-1800 cm^-1^ region. The highlighted areas represent the standard deviations of the spectra within each category.


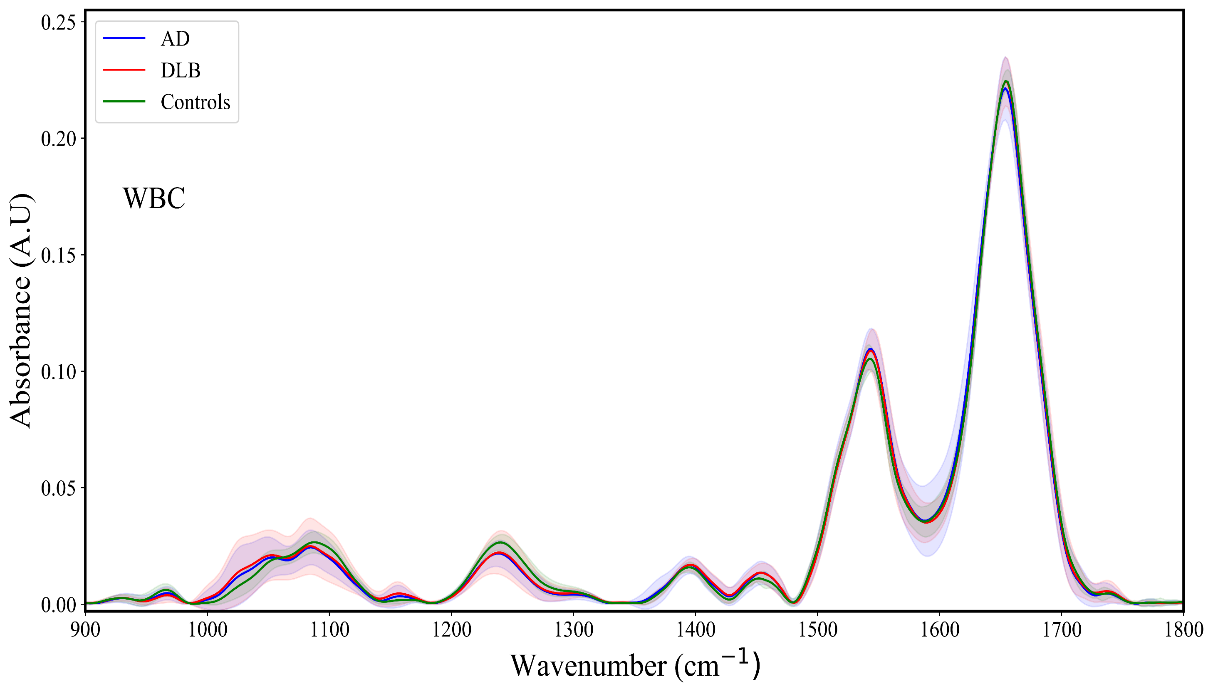


**Fig. 2S:** Plasma IR average spectra of DLB, AD patients, and controls in the 900-1800 cm^-1^ region. The highlighted areas represent the standard deviations of the spectra within each category.


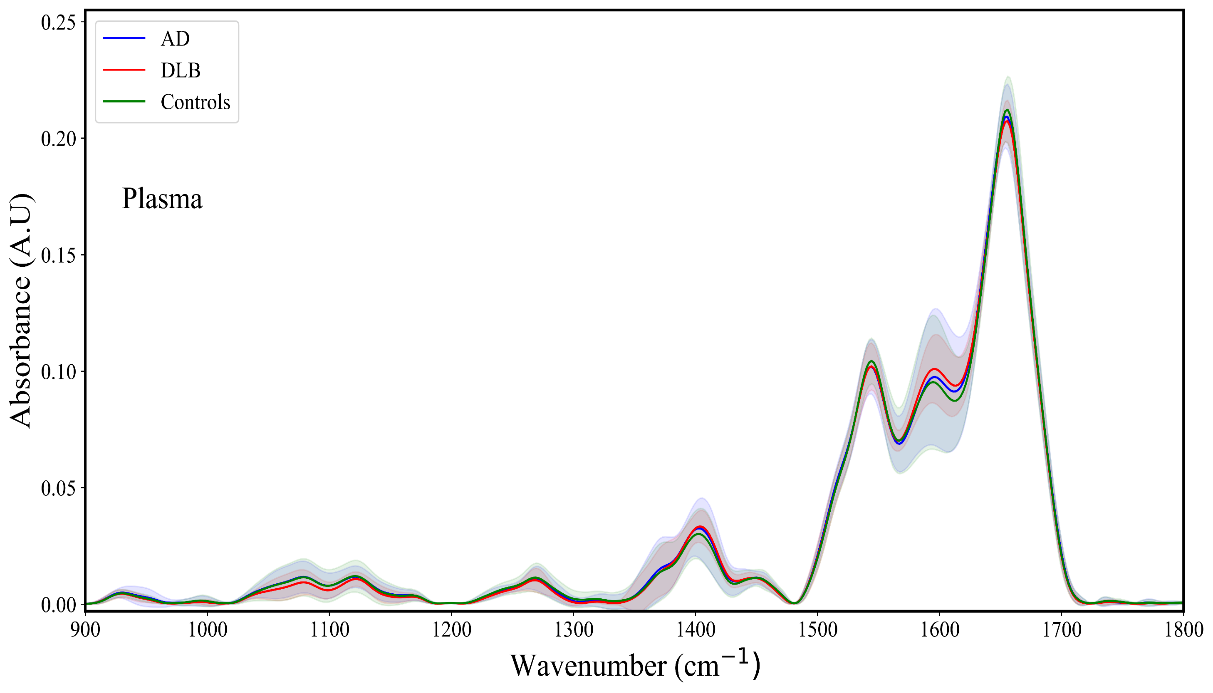


Figure 3S shows the ROC curves for the differentiation between AD and controls using selected features of the second derivative spectra of the two blood components, WBC and plasma separately. The results of the classifications are summarized in Table 3S. Similar analyses were done for the differentiation between DLB and controls (Fig. 4S and Table 4S)

**Figure 3S:** Resulting ROC curves of the different classifiers for the classification between AD and controls categories using selected features from the FTIR second derivative spectra, in the 900-1800 cm^-1^ region, for the two blood components (a) WBC and (b) plasma. The curve scores were derived at the spectrum level using the LOGOCV approach for both classifiers.


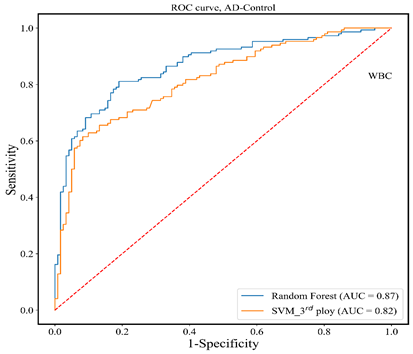

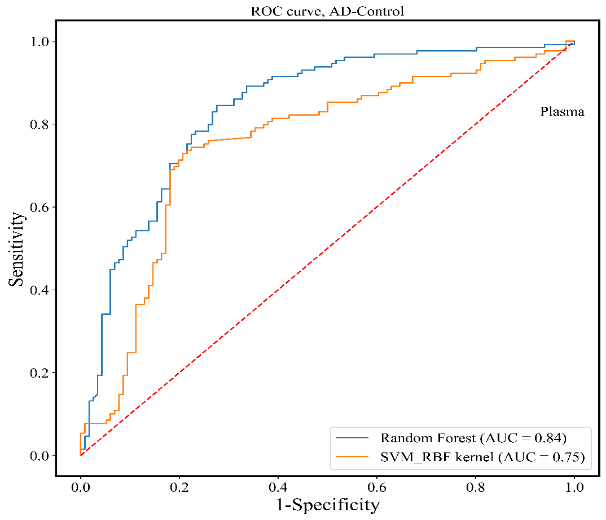


**(a)**

**(b)**

| **Table 3S**: Performances of the best used classifier for the classification between AD and controls categories. The classification results were computed at the patient level by voting the results of the classifier at the spectrum level, derived using the LOGOCV approach, for all the feature vectors that belong to the specific patient. | | | | | | | | | | |
| --- | --- | --- | --- | --- | --- | --- | --- | --- | --- | --- |
|  | Best classifier | AD (No. of spectra) | controls (No. of spectra) | No. of features | SE | SP | Acc | PPV | NPV | AUC |
| WBC | RF | 20 (121) | 26 (145) | 290 | 0.90 | 0.93 | 0.87 | 0.88 | 0.92 | 0.87 |
| Plasma | RF | 18 (87) | 23 (111) | 280 | 0.85 | 0.75 | 0.79 | 0.72 | 0.86 | 0.84 |

**Figure 4S:** Resulting ROC’s curves of the different classifiers for the classification between DLB and controls categories using selected features from FTIR second derivative spectra, in the 900-1800 cm^-1^ region, for the two blood components (a) WBC and (b) plasma. The curves scores were derived at the spectrum level using the LOGOCV approach for both classifiers.


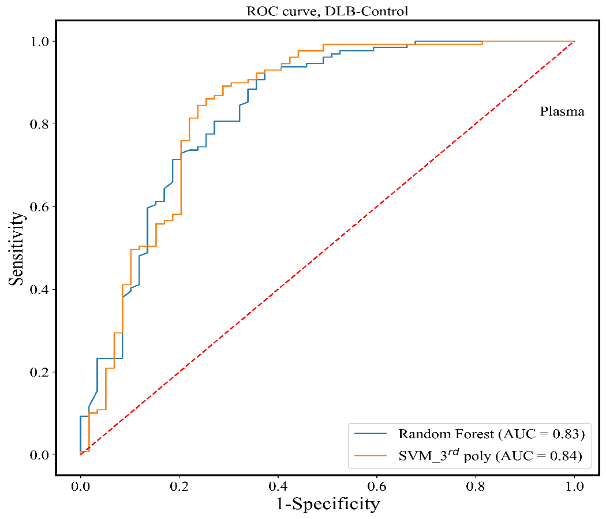

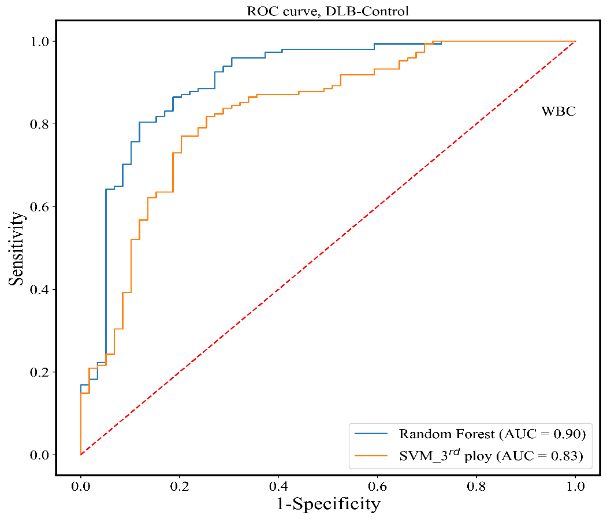


**(b)**

**(a)**

| **Table 4S**: Performances of the best used classifier for the classification between DLB and controls categories. The classification results were computed at the patient level by voting the results of the classifier at the spectrum level, derived using the LOGOCV approach, for all the feature vectors that belong to the specific patient. | | | | | | | | | | |
| --- | --- | --- | --- | --- | --- | --- | --- | --- | --- | --- |
|  | Best classifier | DLB (No. of spectra) | controls (No. of spectra) | No. of features | SE | SP | Acc | PPV | NPV | AUC |
| WBC | RF | 10 (59) | 26 (145) | 280 | 0.90 | 0.81 | 0.83 | 0.64 | 0.95 | 0.90 |
| Plasma | SVM 3^rd^ Poly | 8 (39) | 23 (111) | 440 | 0.85 | 0.83 | 0.84 | 0.63 | 0.94 | 0.84 |

**Fig. 5S:** Displays five pre-processed spectra acquired from five different sites of the same WBC sample of one of the AD patients indicating the high reproducibility of the measurements.


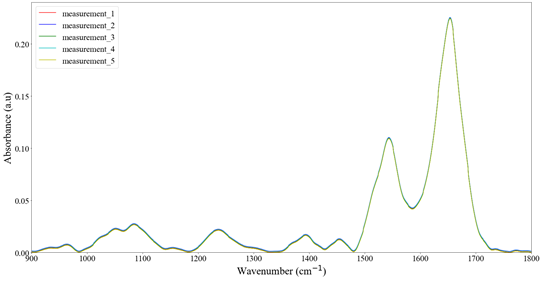

Supplement: Supplementary file 1 [file JBO_025_046501_SD001.docx]
